# Under-recognition of autoimmune polyendocrine syndromes in T1DM: results from an extended screening workup

**DOI:** 10.1007/s12020-026-04716-2

**Published:** 2026-08-01

**Authors:** Valerio Velardi, Lidiana Sanson, Barbara Toffoli, Federico Favaro, Fabio Fischetti, Bruno Fabris, Riccardo Candido, Stella Bernardi

**Affiliations:** 1 Department of Medical Surgical and Health Sciences, University of Trieste, Cattinara Teaching Hospital, Strada di Fiume 447, Trieste, 34149 Italy; 2 SC Patologie Diabetiche, ASUGI (Azienda Sanitaria Universitaria Giuliano Isontina), Via Sai 7, Trieste, 34128 Italy; 3 SS Endocrinologia (Medicina Clinica), ASUGI (Azienda Sanitaria Universitaria Giuliano Isontina), Cattinara Teaching Hospital, Strada di Fiume 447, Trieste, 34149 Italy

**Keywords:** Type 1 diabetes, Autoimmune thyroid disease, Celiac disease, Autoimmune polyendocrine syndromes, Autoimmunity, Screening

## Abstract

**Aims:**

Type 1 diabetes mellitus (T1DM) is frequently associated with other autoimmune disorders, particularly autoimmune thyroid disease (AITD) and celiac disease. Guidelines recommend screening for thyroid and celiac disease in T1DM patients. Nevertheless, in case of their positivity, indicating the presence of an autoimmune polyendocrine syndrome (APS), there is a lack of guidance on how to proceed. Should we screen these patients for other autoimmune diseases? This study’s aim was to evaluate the prevalence of additional autoimmune conditions in a cohort of T1DM patients with AITD and/or celiac disease.

**Methods:**

This cross-sectional study includes adult T1DM patients identified through electronic medical records. Patients with concomitant AITD or celiac disease were invited to undergo an extended screening workup for autoimmune disorders.

**Results:**

639 T1DM patients were identified, and 198 (31%) met the clinical criteria for APS (T1DM plus AITD and/or celiac disease), but only 17 (8.6%) had a formal APS diagnosis in their records. After the extended screening in 139/198 patients, 39% exhibited previously unknown autoantibody positivity, and 11.5% had new autoimmune diseases. Overall, among the patients who underwent the extended screening, 32% (44/139) were diagnosed with at least three autoimmune diseases.

**Conclusions:**

Our study shows that APS is often under-recognized and rarely formally documented in patients with T1DM. In addition, 32% of T1DM patients with AITD or celiac disease have at least a third autoimmune disease. These findings highlight that the identification of AITD or celiac disease in T1DM marks only one stage of the diagnostic journey. APS recognition should lead to a formal diagnosis and ensure heightened clinical vigilance with tailored evaluation of further autoimmune comorbidities during lifelong patient follow-up.

## Introduction

Type 1 diabetes mellitus (T1DM) is frequently associated with other autoimmune disorders, particularly autoimmune thyroid disease (AITD) and celiac disease [1–3], forming autoimmune polyendocrine syndromes (APS) [4]. APS are heterogeneous conditions characterized by the immune-mediated dysfunction of at least two endocrine glands [4]. They include rare monogenic varieties, such as APS-1 (or APECED), caused by AIRE gene mutations and manifesting in youth with candidiasis, hypoparathyroidism, and Addison’s disease [4]. Conversely, polygenic APS types 2–4 are more common adult-onset forms (peaking between 20 and 60 years) with complex environmental and multi-locus genetic susceptibility (e.g., HLA, PTPN22, CTLA-4, MICA) [5, 6].

International guidelines recommend regular screening for thyroid and celiac disease in T1DM patients [7–9]. However, evidence-based guidance on whether testing positive for these conditions should prompt broader screening for further subclinical autoimmunities is lacking. In other words, whether the identification of these conditions should prompt systematic screening for additional subclinical autoimmunities remains an open question. In order to address this research gap, here we performed an extended screening workup in T1DM patients with AITD or celiac disease, in order to evaluate the presence of other autoimmune conditions. In addition, we evaluated whether the presence of APS and the number of autoimmune diseases were associated with glycemic control.

## Materials and methods

This is a real-world cross-sectional study, aiming to evaluate the presence of other autoimmune conditions in T1DM patients with AITD and/or celiac disease. Eligible participants were adult T1DM patients (age > 18 years) followed at the ASUGI Diabetes Center (Trieste, Italy), who had AITD and/or celiac disease and provided informed consent to participate in the study. This study was conducted in accordance with the Declaration of Helsinki, and the protocol was approved by our Institutional Review Board (University of Trieste Comitato Etico di Ateneo #136 dated 30/11/2023).

All adult T1DM patients were identified through electronic healthcare records. First, we collected patient data including demographic characteristics, T1DM duration, medication (multiple daily injections, continuous subcutaneous insulin infusion, none), HbA1c, microvascular complications (nephropathy, neuropathy, retinopathy), and the presence of AITD, celiac disease, and/or other autoimmune conditions (e.g. vitiligo). Second, T1DM patients with concomitant AITD or celiac disease were screened for a prior diagnosis of APS. When this diagnosis was missing, patients were informed and the APS diagnosis was formally established. Subsequently, all these patients were invited to undergo an extended screening workup. Patients were fully informed about the purpose of the research and gave their written informed consent for the inclusion in this study.

The screening included thyroid peroxidase (TPO) antibodies, thyroglobulin (TG) antibodies, thyroid stimulating hormone receptor (TSHR) antibodies, tissue transglutaminase (TTG) antibodies, immunoglobulin A, anti-parietal cell antibodies (APCA), 21-hydroxylase (21-OH) antibodies, antinuclear antibodies (ANA; laboratory threshold for positivity is 1:160), rheumatoid factor (RF), serum protein electrophoresis and vitamin B12. General parameters such as sodium, potassium, aspartate and alanine transaminase, creatinine, and full blood count were also included. In cases of antibody positivity, specific clinical, biochemical, or instrumental evaluations were performed to confirm overt disease according to international guidelines (e.g., gastroscopy for autoimmune gastritis, morning cortisol and ACTH for Addison’s disease, or specialized consultations followed by specific tests for rheumatic diseases or other conditions, such as autoimmune hepatitis) [10].

Overall, APS-2 was defined by the coexistence of Addison’s disease with AITD and/or T1DM; APS-3 was defined by the coexistence of T1DM with AITD (excluding Addison’s disease); APS-4 was defined by combinations of T1DM or AITD with other organ-specific autoimmune diseases (excluding Addison’s disease) [6].

All statistical analyses were carried out in the R system for statistical computing (R version 4.1.0 (2021-05-18); R development Core Team, The R Foundation, Vienna, Austria, 2018). Independent group comparisons of continuous variables were performed with the Wilcoxon test. Qualitative variables were compared with the chi-squared test. Logistic regression was used to evaluate independent associations with APS, adjusting for age, sex, T1DM duration, and T1DM treatment (MDI, CSII, none).

## Results

Out of 639 T1DM patients, 198 (31%) met the clinical criteria for APS (T1DM plus AITD and/or celiac disease), but only 17 of them (8.6%) had a formal APS diagnosis documented in their records. T1DM patients with APS were more frequently female and were treated more often with CSII, as shown in Table 1. Logistic regression showed an independent association of APS with female sex (OR 3.17; 95%CI 2.22–4.58; *P* < 0.0001) and with CSII (OR 1.58; 95%CI 1.07–2.33; *P* < 0.02).

**Table 1:** Characteristics of the patients at baseline

Variable		All patients (*n* = 639)	NO APS (*n* = 441)	APS (*n* = 198)	*p* value
Age (years)		53 (38–63)	53 (38—64)	53 (39–63)	0.941
Sex M/F (n; %)		344/295 (53.8–46.2%)	278/163 (63.0–37.0%)	66/132 (33.3–66.7%)	< 0.0001*
Age at diagnosis (years)		26 (15–38)	26 (15–38)	27 (14–39)	0.928
T1DM duration (years)		21 (12–34)	21 (12–34)	21 (11–36)	0.954
HbA1c (%)		7.2 (6.6–7.9)	7.2 (6.6-8.0)	7.2 (6.6–7.7)	0.334
Therapy	CSII MDI None	206/639 (32.3%) 429/639 (67.1%) 4/639 (0.6%)	123/441 (27.9%) 316/441 (71.7%) 2/441 (0.4%)	83/198 (41.9%) 113/198 (57.1%) 2/198 (1.0%)	0.001*
Dose of insulin (UI)		39 (25–53)	40 (25–54)	37 (26–50)	0.356
Neuropathy (n; %)		138/639 (21.6%)	102/441 (23.1%)	36/198 (18.2%)	0.160
Nephropathy (n; %)		63/639 (9.9%)	50/441 (11.3%)	13/198 (6.6%)	0.061
Retinopathy (n;%)		225/639 (35.2%)	166/441 (37.6%)	59/198 (29.8%)	0.060
Urinary albumin (n; %)	Macroalb Microalb Absent	15/639 (2.4%) 57/639 (8.9%) 567/639 (88.7%)	13/441 (2.95%) 45/441 (10.2%) 383/441 (86.85%)	2/198 (1.0%) 12/198 (6.1%) 184/198 (92.9%)	0.068

A total of 139/198 (70%) T1DM patients with APS agreed to undergo the screening workup. In comparing patients who accepted the extended screening (*n* = 139) against those who declined (*n* = 59), no statistically significant differences were observed between the two groups in terms of age, sex distribution, diabetes duration, or recent glycemic control (HbA1c). Of the 139 patients who underwent this extended screening, 39% (55/139) exhibited previously unknown autoantibody positivity. Among these 55 patients, the specific autoantibodies detected included not only ANA (20%, 28/139) and APCA (15%, 21/139), but also 6 patients with RF (4.3%), 5 patients with 21-OH antibodies (3.6%), 4 patients with TPO and TG antibodies (2.9%), and 1 patient with TTG antibodies (0.7%). Following further diagnostic exams, new autoimmune diseases were identified in 11.5% (16/139) of patients. Chronic autoimmune gastritis was the most frequent disease (*n* = 8), and 2 patients had low vitamin B12, followed by AITD (*n* = 4), celiac disease (*n* = 1), Addison’s disease (*n* = 1), rheumatoid arthritis (*n* = 1), as well as systemic lupus erythematosus (*n* = 1) and autoimmune hepatitis (*n* = 1). These two conditions were both suspected based on high ANA titres and general parameters and confirmed following formal clinical and specialist evaluation.

**Fig. 1:**
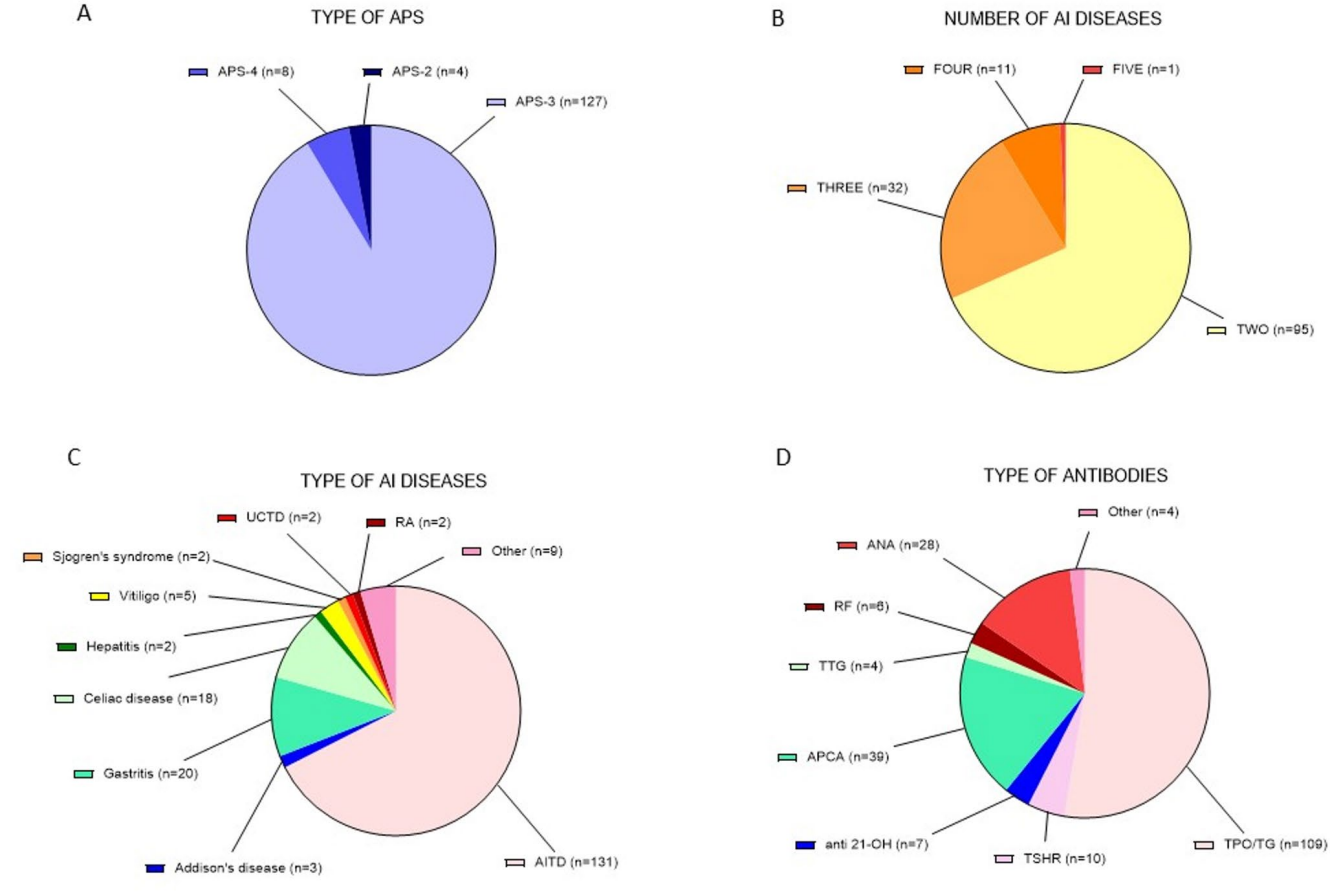
Autoimmune conditions in adult patients with T1DM. (**A**) Type of APS; (**B**) Number of autoimmune conditions in each participant; (**C**) Prevalence of autoimmune conditions; AITD, autoimmune thyroid disease; RA, rheumatoid arthritis; UCTD, undifferentiated connective tissue disease. (**D**) Prevalence of autoantibodies. ANA, Antinuclear Antibodies; Anti 21-OH, Anti-21-Hydroxylase Antibodies; APCA, Anti-Gastric Parietal Cell Antibodies; RF, Rheumatoid Factor; TPO/TG, Thyroid Peroxidase and Thyroglobulin Antibodies; TSHR, Thyroid-Stimulating Hormone Receptor Antibodies; TTG, Tissue Transglutaminase Antibodies

In the end, taking into account both newly diagnosed conditions and those retrieved from medical records, the vast majority of our cohort with APS had APS-3 (91%; 127/139), while APS-4 was present in 5.7% (8/139) and APS-2 was present in 2.9% (4/139), as shown in Fig. 1A. Most importantly, as shown in Fig. 1B, 32% of T1DM patients (44/139) presented with at least three autoimmune diseases. Patients with three or more autoimmune diseases were significantly older than those with two autoimmune diseases, but there were no differences in terms of glycemic control or diabetic complications. As shown in Fig. 1C, the most prevalent condition was AITD (94%; 131/139), including Graves’ disease in 13.6% of cases. Other frequent conditions were autoimmune gastritis (14%), celiac disease (13%), vitiligo (3.6%), and Addison’s disease (2.2%). A prevalence of 1.4% was observed for autoimmune hepatitis, rheumatoid arthritis, undifferentiated connective tissue disease, and Sjögren’s syndrome, while a prevalence of 0.7% was observed for systemic lupus erythematosus, antiphospholipid syndrome, systemic scleroderma, alopecia, and hypoparathyroidism. Figure 1D shows the prevalence of autoantibodies (newly detected and present in the records): TPO and/or TG 109/139 (78.4%), ANA 28/139 (20%), APCA 39/139 (28%), TSHR 10/139 (7.2%), RF 6/139 (4.3%), 21-OH 7/139 (5%), TTG 4/139 (2.9%), AMA 2/139 (1.4%), anti-CCP 1/139 (0.7%); anti-U1rnp 1/139 (0.7%). It should be noted that the discrepancy between clinical diagnoses, which were mostly retrieved from medical records, and autoantibody prevalence may be attributed to several factors: firstly, the presence of seronegative variants, such as seronegative thyroiditis, and secondly, certain conditions may show antibody negativity over time due to effective management, such as a strict gluten-free diet in celiac patients.

## Discussion

Our data show that in a cohort of adult T1DM patients, nearly one-third had AITD or celiac disease. Although 198/639 (31%) met the clinical criteria for APS (T1DM plus AITD and/or celiac disease), only 8.6% had a formal APS diagnosis documented in their records. This illustrates that in current medical practice, APS remains significantly under-diagnosed, which can have a negative impact on patient care. Awareness that a patient has APS may improve their management and outcomes by increasing medical attention to clues of new endocrine or systemic autoimmune diseases resulting in earlier referral to other specialists and earlier diagnosis.

When we compared the clinical characteristics of T1DM patients with and without APS, we found that APS was independently associated with female sex and CSII. The independent association between female sex and APS is consistent with the established higher risk of autoimmune diseases in female subjects [11, 12], which can be attributed to sex hormones, immune genes on the X chromosome, and sex/gender-specific epigenetic effects of estrogen and the environment [11]. In this study, we did not find any difference in glycemic control between T1DM with and without APS, consistent with recent works [13, 14]. However, there was an independent association between APS and the use of CSII. Although this might suggest that an autoimmune disease could affect glucose variability and require a CSII, this finding should be interpreted with caution, as the observational design of the study does not allow causal inference. An alternative explanation is that patients with multiple autoimmune conditions receive a higher level of diabetes care or closer follow-up.

Following the extended screening workup, 39% of patients exhibited previously unknown autoantibody positivity, which led us to identify new autoimmune diseases in 11.5% of patients. The fact that autoantibodies were present even in the absence of an autoimmune disease is consistent with the concept that the production of autoantibodies precedes the development of organ or systemic disease and can help identify patients at risk, such as in Addison’s disease [15]. In addition, it is well known that 10% of healthy individuals have ANA at 1:80 and 5% have ANA at 1:160 [16]. Although ANA positivity has a low predictive value [16], these antibodies should be taken into account in case of high titres (> 1:320). For instance, in our cohort, ANA at high titres (> 1:320) were instrumental in diagnosing one case of systemic lupus erythematosus and one case of autoimmune hepatitis.

In the end, taking into account these new diagnoses and those retrieved from medical records, 32% T1DM patients (44/139) had at least three autoimmune diseases. These findings highlight that the identification of AITD or celiac disease in T1DM should mark only one stage of the diagnostic journey. APS recognition should lead to a formal diagnosis and ensure heightened clinical vigilance with tailored evaluation of further autoimmune comorbidities during lifelong patient follow-up. This is consistent with the suggestion by Dittmar et al. [17] that in T1DM patients, the presence of AITD or celiac disease should prompt for a broader evaluation of additional autoimmune diseases.

This study provides a real-world perspective on the prevalence of autoimmune conditions in a large, well-characterized cohort of adult patients with T1DM followed at a single tertiary center. By focusing on patients who already met the criteria for APS (T1DM plus AITD and/or celiac disease), we were able to quantify the “clinical gap” between the actual prevalence of APS and its formal diagnosis in clinical records. Furthermore, the systematic screening for additional autoimmune conditions allowed us to identify several cases of undiagnosed conditions and assess the overall APS burden. The study’s main limitation is the cross-sectional design, which prevents determining the rate of progression from seropositivity to overt disease. In addition, a portion of the eligible patients (*n* = 59; 30%) did not consent to the additional screening, which may introduce a selection bias. On the other hand, though, epidemiological literature confirms that a 70% participation rate represents a good benchmark in clinical screening and participation rate alone does not introduce selection bias; rather, bias occurs if non-participation is driven by factors systematically associated with the clinical outcomes [18]. Last, our screening was primarily based on antibody detection, which misses seronegative conditions [19].

In conclusion, our study shows that APS is often under-recognized and rarely formally documented in patients with T1DM. In addition, 32% of T1DM patients with AITD or celiac disease have at least a third autoimmune disease. These findings highlight that the identification of AITD or celiac disease in T1DM should mark only one stage of the diagnostic journey. APS recognition should lead to a formal diagnosis and ensure heightened clinical vigilance with tailored evaluation of further autoimmune comorbidities during lifelong patient follow-up.

## Data Availability

The raw data supporting the conclusions of this article will be made available by the authors upon reasonable request.

## References

[1] T.M. Triolo, T.K. Armstrong, K. McFann, L. Yu, M.J. Rewers, G.J. Klingensmith, G.S. Eisenbarth, J.M. Barker, Additional autoimmune disease found in 33% of patients at type 1 diabetes onset. Diabetes Care. **34**, 1211–1213 (2011). 10.2337/dc10-1756 21430083 10.2337/dc10-1756 PMC3114477

[2] J.M. Barker, Clinical review: Type 1 diabetes-associated autoimmunity: natural history, genetic associations, and screening. J. Clin. Endocrinol. Metab. **91**, 1210–1217 (2006). 10.1210/jc.2005-1679 16403820 10.1210/jc.2005-1679

[3] S. Makimattila, V. Harjutsalo, C. Forsblom, P.H. Groop, G. FinnDiane Study, Every Fifth Individual With Type 1 Diabetes Suffers From an Additional Autoimmune Disease: A Finnish Nationwide Study. Diabetes Care. **43**, 1041–1047 (2020). 10.2337/dc19-2429 32139386 10.2337/dc19-2429

[4] E.S. Husebye, M.S. Anderson, O. Kampe, Autoimmune Polyendocrine Syndromes. N Engl. J. Med. **378**, 1132–1141 (2018). 10.1056/NEJMra1713301 29562162 10.1056/NEJMra1713301 PMC6007870

[5] C. Betterle, C. Dal Pra, F. Mantero, R. Zanchetta, Autoimmune adrenal insufficiency and autoimmune polyendocrine syndromes: autoantibodies, autoantigens, and their applicability in diagnosis and disease prediction. Endocr. Rev. **23**, 327–364 (2002). 10.1210/edrv.23.3.0466 12050123 10.1210/edrv.23.3.0466

[6] C. Betterle, J. Furmaniak, C. Sabbadin, C. Scaroni, F. Presotto, Type 3 autoimmune polyglandular syndrome (APS-3) or type 3 multiple autoimmune syndrome (MAS-3): an expanding galaxy. J. Endocrinol. Invest. **46**, 643–665 (2023). 10.1007/s40618-022-01994-1 36609775 10.1007/s40618-022-01994-1

[7] American Diabetes Association Professional Practice Committee for, D. 4, Comprehensive Medical Evaluation and Assessment of Comorbidities: Standards of Care in Diabetes-2026. Diabetes Care. **49**, S61–S88 (2026). 10.2337/dc26-S004 41358897 10.2337/dc26-S004 PMC12690184

[8] E. Frohlich-Reiterer, N.S. Elbarbary, K. Simmons, B. Buckingham, K.N. Humayun, J. Johannsen, R.W. Holl, S. Betz, F.H. Mahmud, ISPAD Clinical Practice Consensus Guidelines 2022: Other complications and associated conditions in children and adolescents with type 1 diabetes. Pediatr. Diabetes. **23**, 1451–1467 (2022). 10.1111/pedi.13445 36537532 10.1111/pedi.13445

[9] AMD-SID, Standard italiani per la cura del diabete mellito. 2018

[10] M.P. Hansen, N. Matheis, G.J. Kahaly, Type 1 diabetes and polyglandular autoimmune syndrome: A review. World J. Diabetes. **6**, 67–79 (2015). 10.4239/wjd.v6.i1.67 25685279 10.4239/wjd.v6.i1.67 PMC4317318

[11] D. Fairweather, D.J. Beetler, E.J. McCabe, S.M. Lieberman, Mechanisms underlying sex differences in autoimmunity. J. Clin. Investig. **134** (2024). 10.1172/JCI180076 10.1172/JCI180076 PMC11405048 39286970

[12] G.J. Kahaly, L. Frommer, Polyglandular autoimmune syndromes. J. Endocrinol. Invest. **41**, 91–98 (2018). 10.1007/s40618-017-0740-9 28819917 10.1007/s40618-017-0740-9

[13] A.M. Lopes, A.R. Leite, P. Ferreira, I. Meira, J. Menino, M. Lourenco, J. Lagoa, B. Viveiros, M.J. Barbosa, S.S. Monteiro et al., Prevalence of autoimmune comorbidities and association with glycemic control by CGM-derived parameters in type 1 diabetes. Endocrine. **90**, 507–516 (2025). 10.1007/s12020-025-04354-0 40663292 10.1007/s12020-025-04354-0

[14] J. Samuelsson, R. Bertilsson, E. Bulow, S. Carlsson, S. Akesson, B. Eliasson, R. Hanas, K. Akesson, Autoimmune comorbidity in type 1 diabetes and its association with metabolic control and mortality risk in young people: a population-based study. Diabetologia. **67**, 679–689 (2024). 10.1007/s00125-024-06086-8 38252314 10.1007/s00125-024-06086-8 PMC10904419

[15] L. Naletto, A.C. Frigo, F. Ceccato, C. Sabbadin, R. Scarpa, F. Presotto, M. Dalla Costa, D. Faggian, M. Plebani, S. Censi et al., The natural history of autoimmune Addison’s disease from the detection of autoantibodies to development of the disease: a long-term follow-up study on 143 patients. Eur. J. Endocrinol. **180**, 223–234 (2019). 10.1530/EJE-18-0313 30608902 10.1530/EJE-18-0313

[16] M. Bagnasco, L. Grassia, G. Pesce, The management of the patient with unexpected autoantibody positivity. Autoimmun. Rev. **6**, 347–353 (2007). 10.1016/j.autrev.2007.01.011 17537379 10.1016/j.autrev.2007.01.011

[17] M. Dittmar, G.J. Kahaly, Polyglandular autoimmune syndromes: immunogenetics and long-term follow-up. J. Clin. Endocrinol. Metab. **88**, 2983–2992 (2003). 10.1210/jc.2002-021845 12843130 10.1210/jc.2002-021845

[18] S. Galea, M. Tracy, Participation rates in epidemiologic studies. Ann. Epidemiol. **17**, 643–653 (2007). 10.1016/j.annepidem.2007.03.013 17553702 10.1016/j.annepidem.2007.03.013

[19] M.V. Lenti, C.M. Rossi, F. Melazzini, M. Gastaldi, S. Bugatti, M. Rotondi, P.I. Bianchi, A. Gentile, L. Chiovato, C. Montecucco et al., Seronegative autoimmune diseases: A challenging diagnosis. Autoimmun. Rev. **21**, 103143 (2022). 10.1016/j.autrev.2022.103143 35840037 10.1016/j.autrev.2022.103143

